# Contrasting Similar Words Facilitates Second Language Vocabulary Learning in Children by Sharpening Lexical Representations

**DOI:** 10.3389/fpsyg.2021.688160

**Published:** 2021-07-06

**Authors:** Peta Baxter, Mienke Droop, Marianne van den Hurk, Harold Bekkering, Ton Dijkstra, Frank Leoné

**Affiliations:** ^1^Donders Centre for Cognition, Donders Institute for Brain, Cognition and Behaviour, Radboud University Nijmegen, Nijmegen, Netherlands; ^2^Behavioural Science Institute, Radboud University Nijmegen, Nijmegen, Netherlands

**Keywords:** second language learning, vocabulary, lexical representations, representational specificity, language instruction, contrasting

## Abstract

This study considers one of the cognitive mechanisms underlying the development of second language (L2) vocabulary in children: The differentiation and sharpening of lexical representations. We propose that sharpening is triggered by an implicit comparison of similar representations, a process we call contrasting. We investigate whether integrating contrasting in a learning method in which children contrast orthographically and semantically similar L2 words facilitates learning of those words by sharpening their new lexical representations. In our study, 48 Dutch-speaking children learned unfamiliar orthographically and semantically similar English words in a multiple-choice learning task. One half of the group learned the similar words by contrasting them, while the other half did not contrast them. Their word knowledge was measured immediately after learning as well as 1 week later. Contrasting was found to facilitate learning by leading to more precise lexical representations. However, only highly skilled readers benefitted from contrasting. Our findings offer novel insights into the development of L2 lexical representations from fuzzy to more precise, and have potential implications for education.

## Introduction

Toward the end of primary school education, most children will have developed a vocabulary of considerable size in their first language (L1). They will be able to read and pronounce a large number of words and know their meanings. At this time, many will also start learning a second language (L2), for which they must acquire new orthographic and phonological word forms and map them onto mostly familiar meanings. This learning process necessarily entails the differentiation and refinement of those foreign lexical representations from less to more precise, not only with respect to phonology, but also orthography and semantics. We propose that a process that triggers the sharpening of representations is implicitly comparing representations to similar ones. We call this representational refinement *contrasting*. In this study, we investigate the process of contrasting and demonstrate that it can effectively be exploited to facilitate L2 word learning by explicitly integrating it in a multiple-choice teaching method.

We will set the stage for our study by first considering to what extent the differentiation process of new words applies to the dimensions of phonology, orthography, and semantics. This will be followed by a discussion on how a foreign-language teaching method that integrates contrasting of similar foreign words may benefit L2 word learning in children. We will then present experimental evidence to show that it can indeed be beneficial under specific circumstances.

Our starting point is the *fuzzy lexicon hypothesis*, according to which the phonolexical and/or phonological representations of newly learned L2 words are initially underspecified, or *fuzzy* (Cook, 2012; Cook and Gor, 2015; Cook et al., 2016; Lancaster and Gor, 2016). This fuzziness leads to inaccuracies in auditory speech perception. One source of errors is that learners may not accurately perceive phonemes that do not exist in their native language. For example, a Dutch-speaking learner of English may in this way confuse *thin* ['θin] with *fin* ['fin], because the phoneme /θ/ does not exist in Dutch. Another source of errors is that L2 learners often rely on phonological information to decide on the meaning of a word. In the absence of semantic or orthographic knowledge about the word *spider*, they might for example use phonological similarity with other known words to erroneously conclude that the word is semantically related to *spy* or *spiral*. As proficiency increases, and more similar words are encountered, representations gradually become more specified.

The fuzzy lexicon hypothesis has predominantly been investigated for L2 phonological processing in adults. Given the overall prominent role of phonology in the development of linguistic skills (see van Goch, 2016; Janssen, 2017), and the fact that phonological representations are crucial to the development of stable representations in memory (Baddeley, 1992; Baddeley et al., 1998), it is unsurprising that it has been the natural starting point for much of the research on the nature of novel L2 representations. However, the notion of a gradual specification process from “holistic” to detailed knowledge and representations also pervades native language acquisition research in children and beyond the phonological dimension. Several theories here posit a gradual sharpening process across linguistic dimensions, such as the lexical tuning hypothesis (Castles et al., 2007), the lexical restructuring hypothesis (Metsala, 1997; Metsala and Walley, 1998), the psycholinguistic grain size theory (Ziegler and Goswami, 2005), and the lexical quality hypothesis (Perfetti and Hart, 2002; Perfetti, 2007).

Many of these theories are concerned with the process of learning to read (e.g., the lexical quality hypothesis), which differs from acquiring phonology or semantics because reading has to be explicitly taught. Nevertheless, similar gradual processes are at play in that learners may initially rely on a fuzzy perceptual representation to recognize a printed word such as *salt*, but their orthographic representations must become more precise when they encounter the word *slat*. By comparing *salt* and *slat*, learners can obtain more precise letter position information necessary for accurate visual word recognition (Grainger and Van Heuven, 2004). Similar considerations apply not only to reading in the L1, but also in the L2. Since reading, including the processing of orthographic input, is a guided process, it is a particularly interesting dimension for considering the effect of specific instruction methods, as in the present study.

This gradual specification process can also be seen during the development of L1 lexical-semantic representations. As for L1 phonological representations, it is an implicit process in native language acquisition. During early language development, cross-situational co-occurrences of semantically related words enable children to associate a word form with the correct concept (Smith and Yu, 2008; Suanda et al., 2014). When they first hear the word *dog* while seeing a dog and a cat, they might generalize the word *dog* to either of these concepts. As the word *dog* is encountered in more contexts, the association between *dog* and the correct animal is updated, becoming more detailed and specific (Clark, 2004).

The development of both orthographic and semantic representations is undoubtedly linked to phonology. Orthographic processing is interrelated with phonological (but also semantic) processing, as models like the Dual Route Cascaded model (Coltheart et al., 2001) attest. Learning how to read involves mapping known sounds onto graphemes, during which phonological information is automatically activated (Frost, 1998). Learning the meaning of words requires children to distinguish speech sounds. In addition, phonology is sometimes required to disambiguate word meaning, for instance to determine whether “read” is in the present or past tense (pronounced ['ri:d] or ['rεd], respectively).

Nevertheless, there is also evidence of cases where other lexical dimensions can overrule phonology. For example, children have been shown to be able to use sublexical orthography to infer the meaning of words without phonology mediating the process (Nation and Cocksey, 2009). Similarly, when encountering new words in the L2, learners may not always have complete or even accurate information about the associated L2 phonology, for instance, when they are learning from word lists or are reading. Even when they do, special items such as homophones require consultation of orthography or semantics to learn the word correctly. For example, because *bawl* ['bɔ*l*] and *ball*[′*bO*l] are pronounced the same; their difference in meaning is signaled by their spelling, necessitating precise orthographic knowledge. Even when L2 learners are acquiring non-special words, they need to acquire specific knowledge of the meaning, and build precise links between form and meaning in order to not confuse words with related concepts. Therefore, drawing on the lexical quality hypothesis (Perfetti and Hart, 2002; Perfetti, 2007), L2 word competency depends on the specificity of not only phonological representations, but also orthographic and semantic representations. A poor sharpening of semantic and orthographic representations may thus also be a contributing factor to why even advanced L2 learners still confuse similar words (Llach, 2015).

In sum, a development from fuzzy to more specified lexical representations during L2 learning is crucial not only when developing novel phonological representations, but for orthographic and semantic representations as well. We therefore propose and test an extension of the fuzzy lexicon hypothesis to L2 orthographic and semantic dimensions. In addition, we propose an extension of the lexical quality hypothesis to a foreign language. Indeed, as indicated above, successful word retrieval depends on specific and tightly bound representations with respect to all three dimensions of orthography, phonology, and semantics (Perfetti and Hart, 2002; Perfetti, 2007). Before we test this extended theoretical view in an experiment, we consider the processes involved in lexical specification in more detail.

The fuzzy lexicon hypothesis implies that one of the forces that drives representations to become more specific during L2 word learning is *similarity*. Similarity already plays a large role in early native language development: As children encounter more similar words, they detect the statistical regularities in how these words sound, clustering them in similar-sounding “competitor sets” (Ziegler and Goswami, 2005). Doing so enables efficient retrieval, as representations must become increasingly detailed to distinguish them from neighboring similar words. This notion of competitor set is also important in the visual domain for L1 and L2 word reading, where sets of similar words make up neighborhoods of the presented target (van Heuven et al., 1998). This notion underlines the role of similarity as a driving force in sharpening representations. As long as no similar words are encountered, a fuzzy representation is sufficient. Only when similar words enter the lexicon, the representations are driven to become more detailed.

In line with this, we propose that an important process involved in lexical specification is what we describe as contrasting: In order to trigger the sharpening process, learners must (implicitly or, as in our study, explicitly) carefully compare similar representations. Drawing on studies of perceptual learning, contrasting likely involves mechanisms of selective attention that guide attention toward the relevant (i.e., distinctive) information (Goldstone, 1998; Francis et al., 2000; Francis and Nusbaum, 2002), thereby decreasing the perceived level of similarity between items (Adini et al., 2002) and resulting in more specific representations. We propose that contrasting is a learning process essential for theories such as the fuzzy lexicon hypothesis and lexical quality hypothesis, and posit that contrasting and gradual specification processes also take part in L2 learning by children, even though they differ from adults in that they are still developing their native representations.

Assuming that contrasting is indeed a process involved during learning, it becomes important from both a theoretical and practical perspective to investigate whether it can be integrated in a learning method, and thereby influence the efficiency with which representations evolve from fuzzy to more specific.

At first sight, using similar L2 words for testing our theoretical position may seem to be counterintuitive. The reason is that researchers have often observed that the presence of orthographic and semantic similarity between L2 words is problematic for learning. Several studies have shown that semantic similarity between words negatively impacts learning (e.g., Tinkham, 1993; Waring, 1997; Papathanasiou, 2009; Ishii, 2015). Orthographic similarity, though less widely researched, can also negatively impact learning (Laufer, 1988; Llach, 2015). As a consequence, some have warned against teaching words in lexical sets (Nation, 2000). However, a commonality of several of these studies is that they manipulate similarity in terms of lists of words, in which the words are similar or dissimilar. A disadvantage of doing so is that one cannot easily disentangle to what extent differences found are due to list effects. Moreover, learners will also need to learn the similar words eventually, so rather than circumventing the similarity issue by presenting dissimilar lists, as sometimes proposed (Nation, 2000), ideally instruction tools should be able to cope with it.

However, none of these studies take into account the characteristics of the *learning method*. Potentially, using a different method may lead to less confusion between similar words, or even facilitation. For instance, learning similar words in a traditional way such as from word lists typically does not encourage learners to focus their attention on challenging lexical elements. Possibly, if these studies had used methods that encourage learners to carefully compare and contrast similar words, focusing on differences between them, similarity might have turned out to not be a hindrance, perhaps even helpful.

In fact, there is some evidence showing that such tasks may help specify representations. In the phonological dimension, lexical specificity training (Logan et al., 1991; Bradlow et al., 1999) is an example of such a contrasting task. In this task, learners repeatedly contrast difficult or undifferentiated phonological contrasts, which sharpens the boundaries between these contrasts. This method has, for example, proven to be helpful for Japanese learners of English, who conflate the phonemes “r” and “l” into a single phonemic category. Using a similar learning task and tracking learners' eye movements, Llompart and Reinisch (2020) determined that, as posited previously, the benefits of contrasting words containing similar phonemes is due to attention being guided toward the relevant, i.e., distinctive, lexical information. This process enables them to encode this information more successfully. These findings match those in the field of perceptual learning, where studies have shown that contrasting similar visual stimuli subsequently made them easier to differentiate (Adini et al., 2002). Recent studies have also shown that lexical specificity training also facilitates L2 vocabulary learning, both in children and (young) adults (van Goch et al., 2014; Janssen et al., 2015; van de Ven et al., 2018). The training leads to more specific, higher quality phonological representations that make new words easier for learners to retrieve.

Though we are not aware of any studies that consider the effects of contrasting the orthographic or semantic dimension in L2 vocabulary learning, the notion of contrasting as a learning method does appear to be generalizable to other learning domains. For instance, in general learning, researchers looked at the effects of learning with multiple-choice questions that were manipulated in such a way that the distractor answers were all plausible, i.e., similar on a certain level (Little et al., 2012; Little and Bjork, 2015). Compared to conditions where the answers were not all equally plausible, this manipulation led to better learning. These findings suggest that contrasting is not only a cognitive mechanism essential to learning, but can be used as an instrument for learning in various fields.

Contrasting methods may be generally beneficial to learning, but it is also likely that their effects are modulated by certain factors, especially in children. In this study, we consider some of the linguistic factors that may impact learning with contrasting. In general, aspects such as prior L2 knowledge (Elgort et al., 2015) and verbal working memory (Kormos and Sáfár, 2008; Linck et al., 2014) are likely to play a role in how well-children are able to learn new words. Because children are beginning readers with still developing L1 representations, other, more specific, characteristics may also come into play during learning by contrasting.

First, there is vocabulary size, which is a robust predictor of language ability (Lee, 2011) and is intrinsically linked to the quality of representations. Verhoeven and Perfetti (2011) note that vocabulary growth can be seen as the combination of *quantity* and *quality* of word representations. A larger vocabulary implies that many of these representations will be more specific, since more similar words will be known. Therefore, given that children have a smaller vocabulary than adults, they might be particularly good candidates to benefit from contrasting. However, we might observe less of an advantage in children with a relatively large vocabulary.

A second child characteristic is reading skill level. Better readers are both more proficient at decoding word orthography and extracting word meaning (Gough and Tunmer, 1986; Verhoeven and Perfetti, 2011). According to the lexical quality hypothesis, the ability to recognize a word depends on the specificity of the form representation, and the ability to extract the meaning depends on the specificity of the meaning representation and link to form (Perfetti and Hart, 2002; Perfetti, 2007). Consequently, less skilled readers might experience too much confusion from a contrasting task to display a learning advantage. In comparison, skilled readers might be particularly good at detecting the differences between the similar words, thereby benefiting from contrasting most.

**Figure 1:**
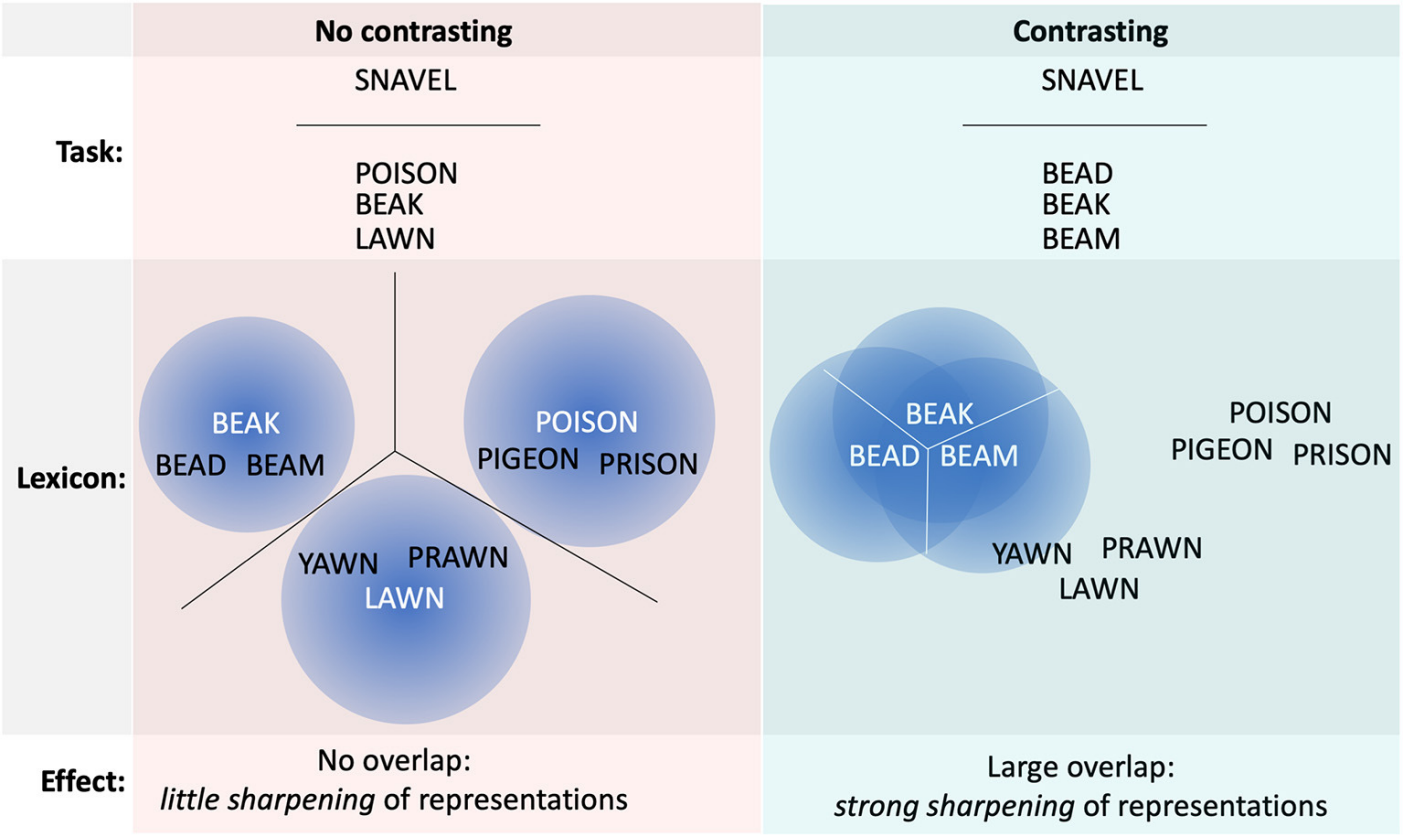
Schematic overview of the expected difference between the no contrasting and contrasting condition. Task shows the used multiple-choice task; Lexicon contains the to-be-learned words, sorted by orthographic similarity in this example; Effect highlights the expected effect on the representations. When not contrasting (left; red), the fuzzy representations (blue gaussian circles) activated for the presented words are not overlapping, hence little sharpening of the representations ensues. When contrasting (right; cyan), the words to be distinguished are largely overlapping, hence the representations need to be sharpened to differentiate the words (white lines).

The primary goal of our study is to determine whether a learning task in which children contrast orthographically and semantically similar L2 words triggers the specification process, consequently facilitating learning. We conducted a visual multiple-choice L2 word learning experiment in which Dutch children learned orthographically and semantically similar English words by either contrasting them or not. The relationship between our task and the specification process is illustrated in Figure 1. Multiple-choice has been shown to be an effective L2 vocabulary learning method (Nakata and Webb, 2016), as it benefits learners by requiring them to practice retrieving the correct answer (see Roediger and Butler, 2011). In the learning task at hand, children saw a Dutch word with three possible English translations, selected an answer, and learned from the feedback. When the answer options were similar, contrasting occurs, as closer comparisons must be made to select the correct options. Word knowledge was then tested with a L2 to L1 translation task at two different points in time.

We hypothesized that contrasting would facilitate L2 word learning by directing learners' attention to relevant lexical information, allowing them to encode this information more precisely, thereby forging more specific representations. In addition, we considered the possibility that the aforementioned linguistic characteristics would moderate the effect of contrasting. In particular, children with a larger vocabulary might benefit from contrasting to a lesser extent than those with a smaller vocabulary; and children with better reading skills might experience a larger contrasting advantage than less skilled readers.

In sum, from a theoretical perspective, determining whether a contrasting learning method is beneficial will further our understanding of how L2 learning results in refined representations for different lexical dimensions and in different participant populations. From a practical perspective, our results may have implications for L2 vocabulary instructions using contrasting as a teaching device.

## Method

### Participants

Fifty-one children in five primary schools in the Netherlands (US grade 4, age range 9–10; 31 boys and 20 girls) participated in the study. They had minimal prior formal English instruction (prior to grade 4 they received a maximum of 30 min per week of informal exposure to English, by listening to songs for example; in grade 4 they received 1 h per week of formal lessons). The study was approved by the Ethics Committee Social Sciences (ECSS) of the Radboud University Nijmegen, and informed consent was obtained from the parents. The pretest revealed that two children knew more than 20% of the to-be-learned words and their data were therefore excluded. In addition, the reading skills scores for one child were not available, therefore they were also excluded from further analysis, resulting in the data of 48 children being analyzed in total.

### Stimuli

The children all learned the same 27 words, namely nine orthographically similar, nine semantically similar, and nine fully dissimilar English words. The Dutch translations were concrete nouns, selected to match in length and frequency across conditions in English. Semantically similar words fell into a common category (e.g., bicycle parts), their semantic relatedness was checked using Snaut (Mandera et al., 2017), an empirically validated online software that calculates the semantic distance between items. Orthographically similar words were selected to be at least 50% similar using normalized Levenshtein distance (Levenshtein, 1966). The fully dissimilar words had little to no orthographic or semantic overlap. The full list of stimuli and their orthographic and semantic similarity can be seen in Appendix 1 (Table A1 for the contrasted condition, Table A2 for the not contrasted condition). To ensure that the children would be familiar with the meaning of the words, we selected Dutch words that are typically acquired earlier than age 9 using (Brysbaert and Biemiller, 2017) age of acquisition (AoA) database. For some words we were unable to find an AoA, to make sure that the meaning would be known we added a picture to each word. Pictures were either retrieved from the Multipic database (Duñabeitia et al., 2018), or were copyright-free images altered to resemble the style of the Multipic pictures.

### Design

#### Word List Structure

In the multiple-choice task, the word list was presented in two distinct ways. One half of the children saw the orthographically and semantically similar words sorted in such a way that they would be contrasted, and the other half not. The fully dissimilar words were presented the same way across conditions, serving as a baseline. This paradigm allowed the exact same words to be learned either contrasted or not, thereby eliminating possible list effects.

Each of the sets of nine words per condition consisted of three groups of three words, which could be combined into three unique triplets of answer options for a single target word. Each triplet was presented once during learning, meaning that each word was seen three times as a target word, and six times as a distractor.

**Figure 2:**
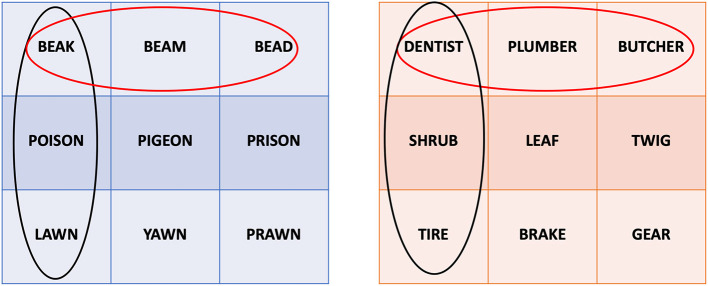
Presentation system allowing for the between-subjects contrasting condition. The blue table on the left contains the orthographically similar words used in the experiment. The orange table on the right contains the semantically similar words used in the experiment. Red circles illustrate example triplets for the contrasted condition, black circles illustrate example triplets for the not contrasted condition. The same principle is applied to all rows and columns.

For the similar words, in the contrasting condition, each group contained three orthographically or semantically similar words, resulting in similar triplets (Figure 2, rows). In the no contrasting condition, one word from each group was picked to create three new groups, resulting in dissimilar triplets with the same words (Figure 2, columns).

For the dissimilar condition, one set of nine fully dissimilar words was selected, resulting in three dissimilar triplets presented once each. For this condition, the children in both the contrasting and the no contrasting condition saw exactly the same triplets during learning.

#### General

The experiment was carried out at five different primary schools in the Netherlands, in relatively quiet rooms. The experimental design consisted of three sessions containing a pretest, learning phase, and posttest, which was repeated a week later as a retention test. In the first session, we administered the pretest. In the second session, which took place at least 1 day later, children carried out the learning phase and immediate post-test. In the third session, exactly 1 week after the learning session, they completed the posttest again as a retention test.

### Procedure

The pretest, learning task, and posttest were all programmed in Expyriment (Krause and Lindemann, 2014), and carried out on a Dell laptop (screen size 14 inches, resolution 1920^*^1080 pixels).

#### Pre- and Post-tests

The pre- and post-tests had an identical format. Children saw all of the English words in the list on screen, in random order. The task was to type the Dutch translation. They did not receive any feedback. Spelling mistakes in Dutch were counted as correct. After the experiment was over, after the retention post-test, the experimenters distributed an answer sheet with the correct translations, and shortly debriefed with the children to discuss how the task went and which words had been learned correctly or incorrectly. The pre- and post-tests lasted 5–10 min each on average.

#### Learning Phase

**Figure 3:**
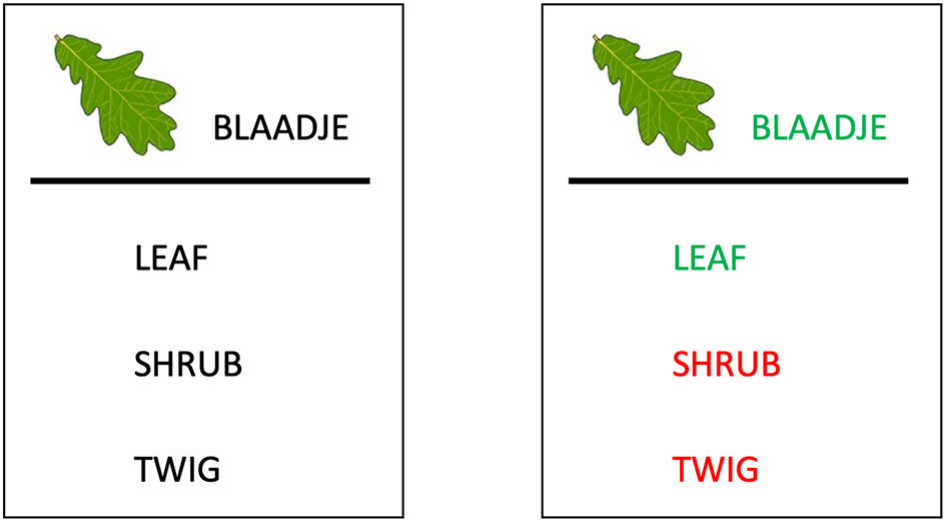
(Left) Example of a trial. At the top, the word in the native language (Dutch), at the bottom three possible English translations. In this example, the words are semantically similar, and in the contrasted condition. (Right) Example of the feedback after children have clicked on an answer. The correct translation pair becomes green, the incorrect answers are red.

The learning phase started with an instruction round in which children were familiarized with the task. The instructions were given on screen through a practice round, and the experimenters also provided oral explanations. Before starting the experiment, the experimenters thoroughly checked whether the task was clear. The children were informed that their classmates would not see the words in the same order as them, and were asked to focus on their own task. In the learning task, children completed three blocks in which each word was presented once. This thus resulted in 81 trials. On each trial, they saw a Dutch word and corresponding picture, with three possible translations aligned vertically below (Figure 3, left). They were instructed to carefully read all answer options, and then click on the translation they thought to be correct. There was no time limit to click on an answer.

Once a word had been clicked, visual feedback was presented for 5 s before the next trial appeared. Feedback was given by highlighting the correct translation and target word in green, while the incorrect answers were highlighted in red (Figure 3, right). This ensured that the feedback was visually identical regardless of whether a mistake had been made or not.

Between each block they saw how many trials they had answered correctly, and were encouraged on screen to try to improve their score. This was included to increase motivation to perform the task. In total, the learning task took ~20 min.

### Additional Measures

In addition to the main experimental components, we also measured covariates that were likely to affect learning or interact with contrasting, namely Dutch vocabulary size, verbal working memory, reading skills, and amount of contact with English.

#### Dutch Vocabulary Size

We measured the children's Dutch vocabulary size using a computerized version of the Dutch Peabody Picture Vocabulary Test (PPVT) (Dunn et al., 2005). In this task, children hear a Dutch word and must indicate to which of four pictures they see on screen it corresponds. Words are clustered in sets of 12 of increasing difficulty. The start set is selected according to the children's age. These sets are then used to calculate the children's raw scores (number of words heard-number of mistakes), which gives an indication of how many words they know. The PPVT has good reliability (0.94; Dunn et al., 2005). We administered the PPVT during the first session, before the pretest, and it took ~20 min.

#### Verbal Working Memory

Verbal working memory was assessed using an adaptation of the Dutch 15-Woordentest (15-WT) (Saan and Deelman, 1986), in which children had to remember a series of 15 auditory words. They heard each series five times, after each time they had to recall all the words they remembered and their score was calculated. We only measured immediate recall to limit the testing time. Their raw scores were the number of words remembered in total. The 15-WT has good reliability (0.80–0.83; Saan and Deelman, 1986). We administered the 15-WT during the first session, after the PPVT, and it took ~10 min.

#### Reading Skills

In addition, we also measured the children's reading skills using the Dutch standardized school test “Analyse van Individualiseringsvormen” (AVI) (Krom et al., 2010). This test measures whether children's reading skills by measuring their speed and accuracy while reading short texts of increasing difficulty. Their score consists of the grade their reading skills correspond to. This can be on par with their current grade, or grades above or below their current grade. In our study, this resulted in 9 possible scores (1 = middle of US grade 2; 9 = beyond the end of US grade 5; with a score of 5 corresponding to a level equivalent to their current grade). The reliability of this test is good (0.94–0.97; Krom et al., 2010). This test was administered by the teachers at the end of the previous school year.

#### Contact With English

Finally, we also measured how much contact children had with written and spoken English outside of school by means of a questionnaire. This questionnaire consisted of 7 questions, which included questions such as “How often do you watch films in English?” The outcome of the contact with English questionnaire was a value between one and four (1 = no contact with English, 4 = a lot of contact with English). The full questionnaire translated to English can be found in Appendix 2. We administered this questionnaire at the beginning of the third session, and children took ~5 min to complete it.

## Results

### Data Analysis

To determine whether contrasting facilitated learning in the different similarity conditions, as well as the effect of the covariates, we conducted generalized linear mixed-effects regression models using the lme4 package in R (Bates et al., 2014). Following the recommendations of Meteyard and Davies (2020), the models' parameters selection was driven by the research question, and we thus only included relevant interactions, which also helps avoid overfitting issues.

The learning phase and post-tests data were analyzed separately. In the learning model, the fixed factors were block (as a continuous factor), word similarity (orthographically similar / semantically similar / fully dissimilar), contrasting (contrasted / not contrasted), and the interaction between contrasting and similarity. In the post-tests model, the fixed factors were time of testing (immediate/retention), word similarity, contrasting, and the interactions between contrasting and time of testing, and contrasting and word similarity. Accuracy both in the learning phase and the post-tests was measured binarily.

For the similarity variable, we used Helmert contrasts to compare the effect of semantic similarity vs. orthographic similarity, and similar words (orthographically + semantically) vs. dissimilar words.

All additional measures (i.e., PPVT scores, AVI scores, 15-WT scores, and contact with English) were added as covariates to the models. The PPVT and 15-WT data were rescaled by z-score normalization and centered around the mean.

In all models, we added random intercepts for words and subjects nested within school. Nesting the subjects within the schools provides an indirect way of controlling for potential differences in socio-economic status, which can impact educational achievement (Strenze, 2007). We did not add any random slopes, as we had no theoretical reason to believe these would affect the results and would result in an unnecessarily complex set of models.

### Descriptive Statistics

On average, children knew 3% (*SD* = 1.8%) of the words, or approximately one word, before doing the learning task. This word was not consistent across children, but the words “prison,” “smoke,” and “pants” were often known to the children prior to learning. At the end of the last block of the learning phase, they accurately selected 76.9% (*SD* = 42.2%) of the words on average, or ~21 of the 27 words. Immediately after learning, the children were able to recall over one-third of the words (37.6% [*SD* = 48.4%], or ~10 words). One week later, this performance decreased to slightly less than one third (24.6% [*SD* = 43.1%], or ~7 words). As could be expected, there was a large amount of individual variation between the children's performances. The lowest accuracy across posttests was one word (*M*_*acc*_ = 1.9%), while the highest was 19 words (*M*_*acc*_ = 72.2%).

### Learning Phase

The analysis results, in which we considered the effects of word similarity, contrasting, and their interaction, can be seen in Table 1.

**Table 1:** Summary of the generalized linear mixed-effects model for the learning data, including estimates, standard errors (SE), *z* values, and significance level.

**Fixed Effects**				
	**Est/Beta**	**SE**	***z***	***p***
Intercept	0.43	0.44	0.98	N.s.
Ortho vs. semantics	0.17	0.25	0.67	N.s.
Similar vs. dissimilar	0.01	0.25	0.02	N.s.
Contrasting	−0.36	0.19	−1.88	N.s.
**Block**	**0.61**	**0.05**	**13.18**	**<0.001**
**AVI**	**0.14**	**0.03**	**4.01**	**<0.001**
PPVT	0.03	0.09	0.35	N.s.
15-WT	−0.08	0.09	−0.94	N.s.
**English knowledge**	–**0.37**	**0.15**	–**2.44**	**<0.05**
Contrasting * ortho vs. semantics	0.01	0.18	0.03	N.s.
**Contrasting** * **similar vs. dissimilar**	**0.48**	**0.18**	**2.67**	**<0.005**
**Random effects**				
	**Variance**	**S.D**.		
School: subject (Intercept)	0.25	0.50		
Word (Intercept)	0.20	0.45		
**Model fit**				
*R*^2^	**Marginal**	**Conditional**		
	0.10	0.21		

Model equation: accuracy ~ similarity^*^contrasting + *AVI* + *PPVT* + *15-WT* + *English Knowledge* + *(1|school: subject)* + *(1|word)*.

*Bolded values are significant*.

*N.s., Non-significant; Ortho, Orthography; AVI, Reading skills; PPVT, Dutch vocabulary; 15-WT, Verbal memory*.

#### Main Variables

The analysis showed a significant main effect of block, indicating that children learned from the multiple-choice task during the learning phase (*M*_*prop*_*corr*_ = 0.54, *SD* = 0.50; *M*_*prop*_*corr*_ = 0.67, *SD* = 0.47; *M*_*prop*_*corr*_ = 0.77, *SD* = 0.42 for blocks 1, 2, and 3 respectively). There were no other significant main effects.

**Figure 4:**
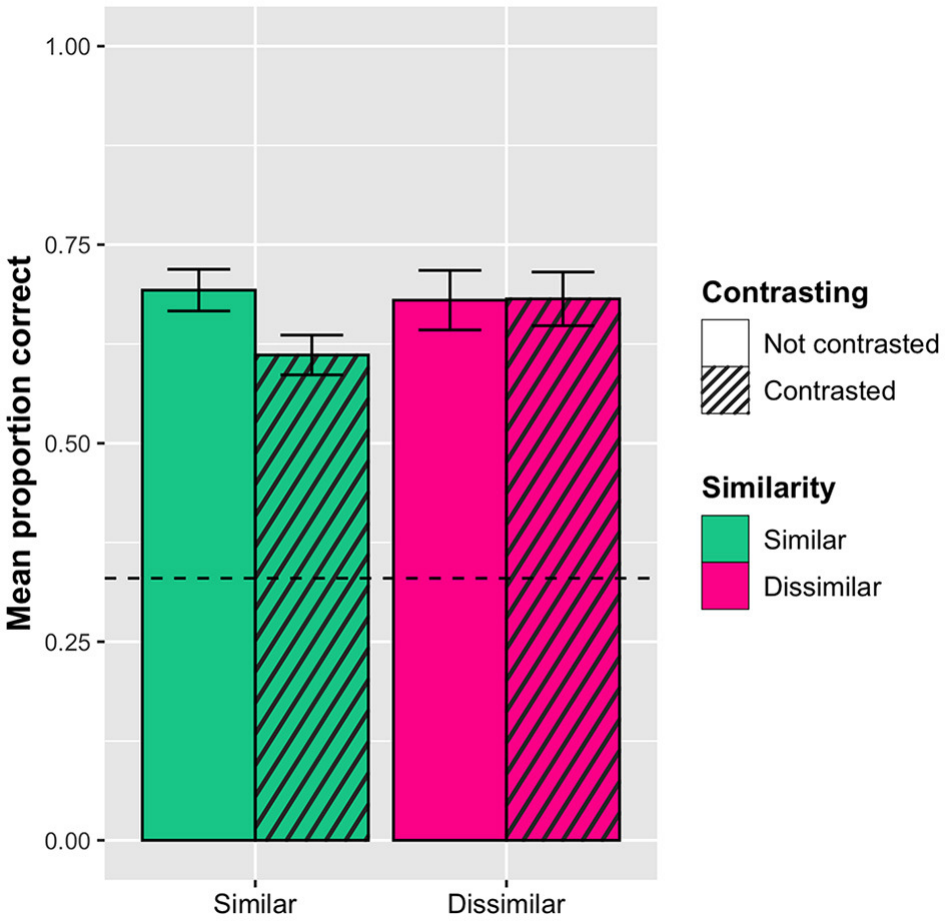
Interaction effect between sorting and similarity (orthographic and semantic similarity collapsed) with 95% confidence interval bars. Dashed line represents chance level (0.33). The similar words are significantly less accurate in the contrasted condition compared to the no contrasting condition.

In addition, the analysis revealed a significant interaction effect between contrasting and similarity, with the similar words (orthographically + semantically) in the contrasted condition being less accurate (*M*_*prop*_*corr*_ = 0.61, *SD* = 0.48) than the similar words in the not contrasted condition (*M*_*prop*_*corr*_ = 0.69, *SD* = 0.46). In contrast, there was no difference for the dissimilar words (*M*_*prop*_*corr*_ = 0.68, *SD* = 0.47 in the contrasted condition; *M*_*prop*_*corr*_ = 0.68, *SD* = 0.47 in the not contrasted condition). In other words, seeing the similar words presented together as distractors in the multiple-choice task made learning them more difficult. This effect can be seen in Figure 4.

#### Additional Measures

**Figure 5:**
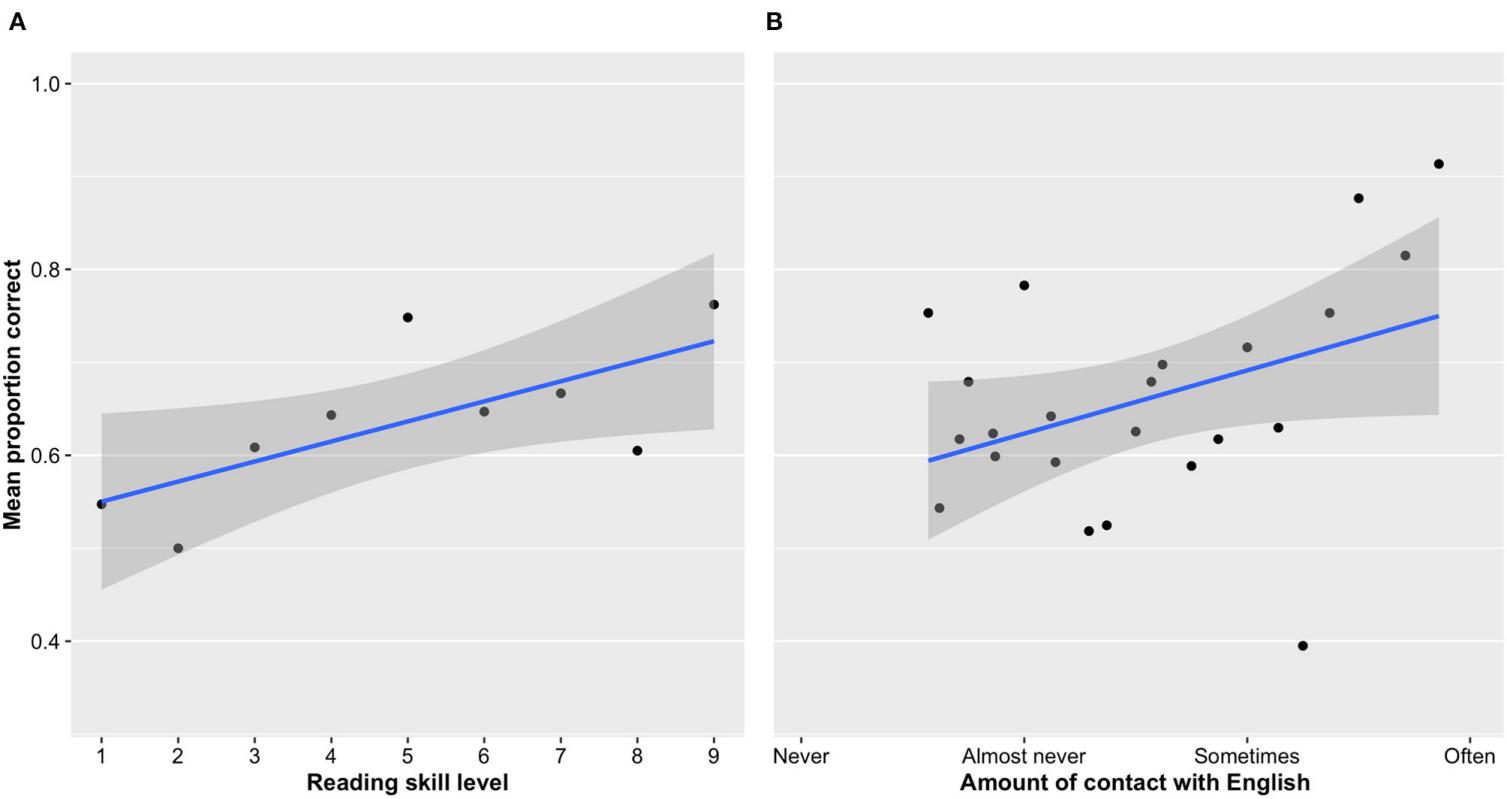
The shaded areas represent the 95% confidence interval. **(A)** Effect of reading skill level on accuracy during learning. Skilled readers learned more words in the multiple-choice task. The dots represent the average proportion of words remembered for all the children within a given level of reading skill **(B)** Effect of knowledge of English on accuracy during learning. Children who had more previous knowledge of English learned more words during the multiple-choice learning task. The dots represent the average proportion of words remembered for all the children within a certain level of English knowledge.

The analysis revealed a strong effect of reading skills as measured by the AVI, with the children with higher reading skills performing better on the learning task than those with lower reading skills (Figure 5A). In addition, children with better knowledge of English also learned better (Figure 5B). The other covariates had no effect on learning.

### Post-tests

The analysis results, which consider the effect of word similarity and contrasting during both the posttest and the retention test can be seen in Table 2.

**Table 2:** Summary of the generalized linear mixed-effects model for the post-tests data, including estimates, standard errors (SE), *z* values, and significance level.

**Fixed Effects**				
	**Est/Beta**	**SE**	***z***	***p***
Intercept	−1.08	0.71	−1.52	N.s.
Ortho vs. semantics	0.14	0.26	0.53	N.s.
Similar vs. dissimilar	0.13	0.15	0.89	N.s.
Contrasting	0.22	0.29	0.75	N.s.
**Time_testing**	**–1.00**	**0.15**	**–6.61**	**<0.001**
**AVI**	**0.23**	**0.06**	**4.06**	**<0.001**
PPVT	−0.04	0.14	−0.30	N.s.
15-WT	−0.04	0.15	−0.30	N.s.
English knowledge	−0.40	0.25	−1.58	N.s.
Contrasting * Ortho vs. semantics	0.03	0.12	0.24	N.s.
Contrasting * Similar vs. dissimilar	**–0.21**	**0.07**	**–2.97**	**<0.005**
Contrasting*Time_testing	0.28	0.20	1.40	N.s.
**Random effects**				
	**Variance**	**S.D**.		
School: subject (Intercept)	0.77	0.88		
Word (Intercept)	1.04	1.02		
**Model fit**				
*R*^2^	**Marginal**	**Conditional**		
	0.11	0.42		

Model equation: accuracy ~ similarity^*^contrasting + block + AVI + PPVT + 15-WT + English Knowledge + (1|school: subject) + (1|word).

*Bolded values are significant. N.s., Non-significant; Ortho, Orthography; AVI, Reading skills; PPVT, Dutch vocabulary; 15-WT, Verbal memory*.

#### Main Variables

Unsurprisingly, analysis of the post-test data revealed a main effect of time, with children knowing more words immediately after learning (*M*_*prop*_*corr*_ = 0.38, *SD* = 0.48) than 1 week later (*M*_*prop*_*corr*_ = 0.25, *SD* = 0.43). There were no other significant main effects.

**Figure 6:**
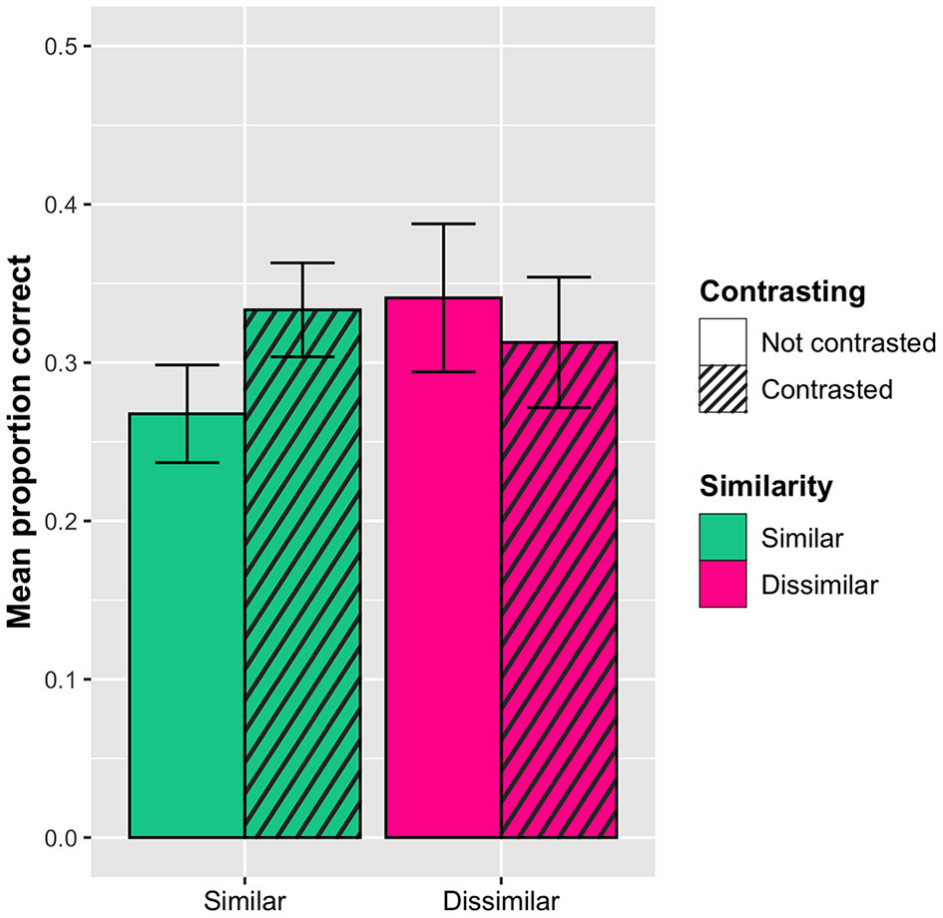
Interaction effect on the post-tests between sorting and similarity (orthographic and semantic similarity collapsed) with 95% confidence interval bars. The similar words are remembered significantly better when they have been contrasted than when they have not.

In addition, the analysis did reveal a significant interaction between word similarity and contrasting: The similar words were remembered better when they had been contrasted (*M*_*prop*_*corr*_ = 0.33, *SD* = 0.47) than when they had not (*M*_*prop*_*corr*_ = 0.27, *SD* = 0.44), whereas there was no difference for the dissimilar words (*M*_*prop*_*corr*_ = 0.31, *SD* = 0.46 in the contrasted condition; *M*_*prop*_*corr*_ = 0.34, *SD* = 0.48 in the not contrasted condition). This effect can be seen in Figure 6.

#### Additional Measures

**Figure 7:**
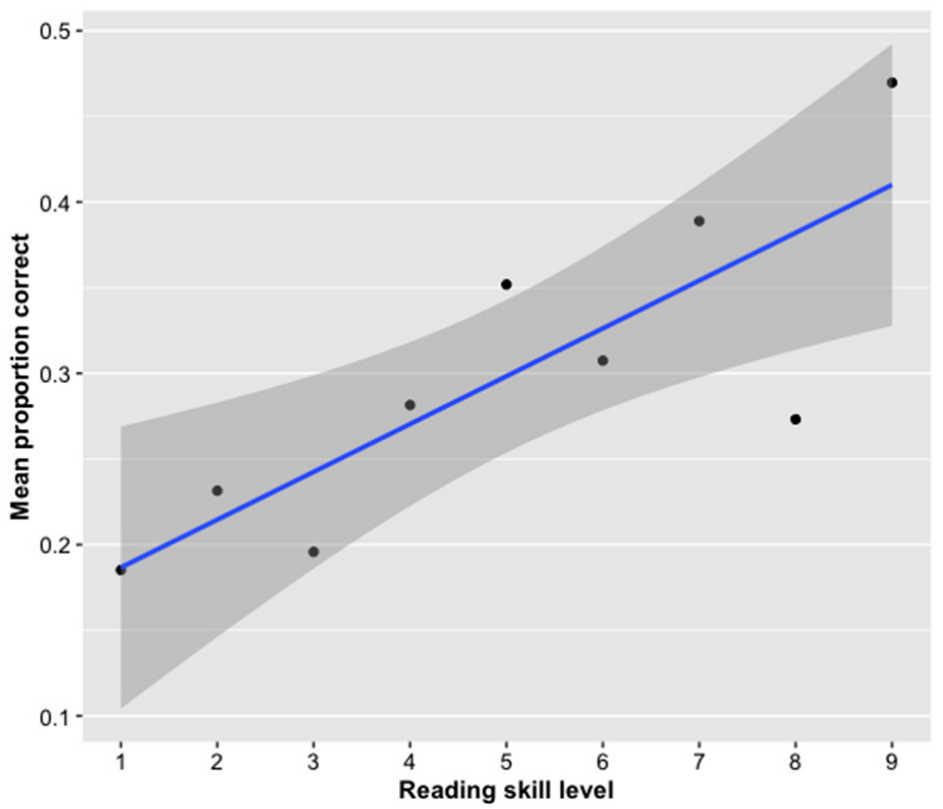
Effect of reading skill level on accuracy in the post-tests. Skilled readers remembered more words than less skilled readers. The dots represent the average proportion of words remembered for all the children within a given level of reading skill. The shaded area represents the 95% confidence interval.

The analysis showed a significant effect of reading skills as measured by the AVI, with children with higher reading skills performing better on the post-tests than those with lower reading skills (Figure 7). The other measures did not explain any of the variance.

### *Post-hoc* Analyses

#### Moderating Role of Reading Skills

Because reading skills appeared to explain a highly significant part of the variance in both the learning phase and the post-tests, we investigated this further in *post hoc* tests. In particular, we investigated whether reading skills moderated the effect of contrasting with multiple regression models. We only modeled the orthographically and semantically similar conditions, since in the dissimilar condition no contrasting occurs.

**Figure 8:**
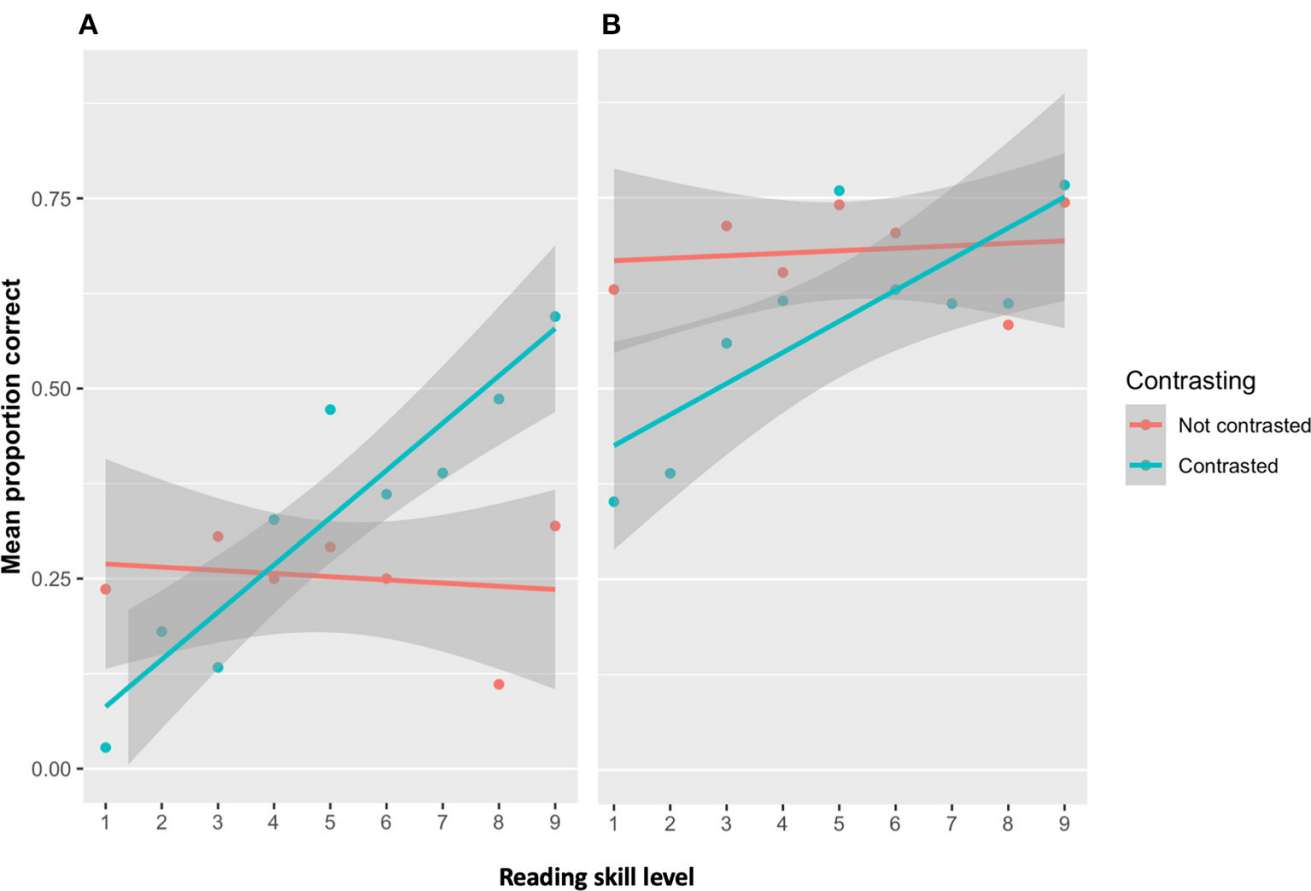
The dots represent the average proportion of words remembered for all the children within a given level of reading skill (red = all children in the not contrasted condition, blue = all children in the contrasted condition). The shaded area represents the 95% confidence interval. **(A)** Effect of contrasting on the similar words during learning depends on reading skill level. Less skilled readers were negatively impacted by contrasting than skilled readers. **(B)** Effect of contrasting on the similar words on the post-tests depends on reading skill level. Contrasting leads to more remembering of the words, but only for skilled readers.

The regression on the learning phase data revealed that reading skills significantly moderated the effects of contrasting [*b* = 0.02, *t*_(3884)_ = 3.49, *p* < *0.001*]. Relative to the not contrasted condition, the performance of children in the contrasted condition more strongly depended on reading skills. Specifically, children with lower reading skills were less accurate during learning than those with higher reading skills (Figure 8A). This indicates that the children with lower reading skills were negatively affected by contrasting during learning, while those with higher reading skills were not.

In the post-tests, the same analysis revealed that reading skills level also significantly moderated the effects of contrasting [*b* = 0.05, *t*_(2, 588)_ = 7.32, *p* < *0.001*]. The children in the contrasted condition with higher reading skills remembered more words than those with lower reading skills. In addition, the confidence intervals (see Figure 8B) indicate that the children in the contrasting condition that had average to above average reading skills (i.e., scores 5–9) remembered more words than all children in the not contrasted condition.

#### Error Characteristics

In order to gain more insight into the data, we also analyzed the children's types of errors on the post-tests, comparing the contrasted to the not contrasted condition. Again, we only considered the similar words. Specifically, we considered cases in which children confused a word for a similar one in the list. We conducted a Wilcoxon signed rank test, which revealed that children in the contrasted condition made significantly more misselections of similar words in the list (e.g., answering the Dutch word for *prison* instead of *poison*) than children in the not contrasted condition (*N* = 63 and *N* = 36, respectively, *W* = 14,430, *p* < 0.05). This suggests that children's representations for these words were more specific than for those for which they provided a dissimilar answer or no answer.

## Discussion

The main goal of this study was to determine whether contrasting orthographically and semantically similar L2 words in a multiple-choice learning task would facilitate children's learning of these words. We proposed that theories such as the fuzzy lexicon hypothesis (Cook et al., 2016) and the lexical quality hypothesis (Perfetti and Hart, 2002; Perfetti, 2007) apply to L2 orthographic and semantic dimensions, and that these theories can be further differentiated by incorporating contrasting as an underlying process involved in specification. In this process, lexical representations evolve from fuzzy to specific, because a sharpening process is triggered when they are contrasted with other, similar, representations. We therefore hypothesized that integrating contrasting in a learning method would facilitate L2 word learning, but that children's linguistic characteristics, such as native vocabulary size and reading skills may mediate this effect.

Our study provides promising initial evidence that contrasting is an effective learning method that has the potential to facilitate L2 word learning in children. After learning orthographically and semantically similar words by contrasting them, children made fewer mistakes when trying to recall these words than children who did not contrast them. These effects persisted a week after learning, which is remarkable given that the learning session only lasted 15 min. Because no differences between contrasting conditions arose for fully dissimilar words, this indicates that contrasting is indeed a process involved in specification, and that our results were not due to a general task effect between groups. As expected, the findings show that the effects of contrasting partly depended on the children's language skills. Specifically, in our study children with lower reading skills were negatively affected by contrasting during learning, but experienced no learning (dis)advantage on later post-tests. Children with higher reading skills were not hindered by contrasting during learning, and recalled significantly more words than all other children on the post-tests.

In the next section, we consider the underlying learning mechanisms in more detail.

### Underlying Mechanisms

As mentioned previously, the sharpening of lexical representations by contrasting most likely involves mechanisms of selective attention (Nosofsky, 1986; Goldstone, 1998; Francis et al., 2000; Adini et al., 2002; Francis and Nusbaum, 2002). In the case of language learning, selectively attending to the lexical dimension in which similarity occurs allows more precise representations to be built, which in turn facilitates L2 word learning because the representations are of higher quality (Perfetti and Hart, 2002; Perfetti, 2007; van de Ven et al., 2018; Llompart and Reinisch, 2020).

In our study, contrasting the similar words explicitly required children to focus on the distinctive lexical information in order to discriminate the correct answer from the distractors. In this way, the task required them to sharpen the boundaries between the novel word representations, thereby boosting how efficiently novel representations are sharpened. Children who did not contrast the similar words, were not stimulated to create as precise representations on a trial-by-trial basis, since the clearly distinct orthography and meaning could be used to identify the correct translation. This is reflected by the finding that children who contrasted were less accurate during learning, but more accurate on the post-tests. The children who did not contrast thus had an easier task during learning, but did not undergo the sharpening process to the same extent. Their initial, fuzzier representations were therefore sufficient to complete the learning task successfully, but when they had to retrieve the correct translation during the post-tests, the impreciseness of the representation led to confusion. This is further supported by the kind of errors children who did not contrast made: They came up with a larger number of translations that were not similar to the correct translation (e.g., translating *beak* with the Dutch word for *poison*). By comparison, children who did contrast the similar words not only recalled more words after learning, but the mistakes they made were orthographically or semantically closer to the correct answer (e.g., translating *prison* with the Dutch word for *poison*). This supports the idea that the children who contrasted developed more precise lexical representations.

Given these findings, it is also possible that contrasting benefited learning because errors were more numerous during learning in this condition. Research has extensively shown that making errors largely benefits (word) learning when feedback is provided, particularly in instances where learners are not confident in their answers (Pashler et al., 2005; Metcalfe and Kornell, 2007; Butler et al., 2008). In addition, making related errors during learning has been shown to lead to better retention of materials (Huelser and Metcalfe, 2012). Since incorrect answers in the contrasted condition were always related (i.e., similar) to the target, this might also have played a role during learning.

Our study also revealed that only skilled readers benefitted from contrasting in the multiple-choice method. Since Dutch (L1) vocabulary size did not impact the effects of contrasting, it is likely that contrasting effects in English (L2) were affected not so much by the initial precision of the Dutch representations, but by fundamental aspects that are required for word reading, such as word decoding and word meaning extraction (Gough and Tunmer, 1986; Verhoeven and Perfetti, 2011). Said differently, for less skilled readers, contrasting the three similar alternatives in the multiple-choice task would have been particularly confusing, making them less efficient at visually teasing the orthographically similar words apart or to differentiate the similar meanings during learning. If reading skill level affects children's efficiency of contrasting, adapting the learning task in certain respects for children with lower reading skills might actually be beneficial. For example, simply making the learning phase longer might affect the outcome of contrasting. In addition, providing explicit cues to attend to certain lexical characteristics could also contribute to an increase of contrasting effectiveness. This latter method has been successfully applied in earlier studies to the orthographic dimension, where children with poor reading skills benefited from a method drawing their attention to the relevant grapheme position in minimal pairs of words (McCandliss et al., 2003).

### Limitations

In our study, we focused on orthographic and semantic representations. However, as we discussed in the introduction, phonology plays an important role in acquiring new words visually. This is especially true for children, who have less well-developed orthographic and semantic representations (Perfetti, 2007; van Goch, 2016; Janssen, 2017; Meade, 2020). While we did not explicitly offer phonological information in our learning task, we assume that children automatically activated phonology when reading the words (Frost, 1998). Therefore, it is likely that a similar contrasting process implicitly occurred on the phonological dimension during the learning task. Given previous findings (e.g., Janssen et al., 2015; van Goch et al., 2017; van de Ven et al., 2018), contrasting phonological information during learning most likely did contribute to the sharpening of the children's lexical representations and possibly created an additive effect. Future research should explore this issue by adding a condition in which the phonetic sounds of the words are made available during learning.

In addition, our results must be interpreted with some caution, as there is a degree of uncertainty with regards to the magnitude of the effects. Because the overall number of learned words was relatively low (befitting the short learning duration), the differences between conditions in terms of words learned is also only a one-word difference (from 5 to 6 words). Percentage-wise, however, the difference translates to a 20% increase relative to all similar words learned. It remains to be determined, though, whether the effects we observed are absolute, i.e., whether when the item sample size is increased the one-word difference would remain the same or scale up correspondingly. If the latter is the case, this would represent significant learning gains potential. In order to determine the actual magnitude of the effects, follow-up studies with increased power in terms of participants and items are required. Additionally, research has shown that a larger number of repetitions positively impact vocabulary learning outcomes (Webb, 2007). Therefore, it is possible that a longer learning phase leads to greater learning gains, particularly for less skilled readers. A longer learning task would allow testing vocabulary knowledge in a more challenging manner, for instance by asking learners to type out the L2 translations of the words. This could offer additional insight into the nature of their lexical representations.

Finally, on a related and practical note, there are some drawbacks to conducting research in ecologically valid settings such as schools, for instance the possibility of distraction during the experiment. However, in our study, we nevertheless obtained significant results in such an environment. While laboratory studies may offer more controlled insights into the mechanisms of contrasting as a learning method, our study highlights its effectiveness in instructional settings.

### Future Directions

Our study has shed light on an important process involved in lexical specification. Contrasting offers several opportunities for future research aimed to gain a more comprehensive understanding of L2 word learning. In particular, it would be valuable to replicate this research with different measuring methods, in order to obtain more detailed insight into the attentional mechanisms underlying contrasting. For instance, to find out more about the lexical aspects learners attend to while they are learning by contrasting, tracking their eye movements would offer valuable additional information (cf. Llompart and Reinisch, 2020).

Furthermore, more work is needed to determine the precise circumstances under which contrasting facilitates learning, in particular in relation to the linguistic contexts and individual characteristics. To gain a fuller understanding of the effects of contrasting in different linguistic settings, future research could explore its effects on different L1-L2 pairings (such as languages with less cross-linguistic similarity), non-alphabetic languages, or even consider the effects of contrasting several languages simultaneously. Moreover, particularly for target learners with linguistic difficulties (e.g., lower reading skills), testing how the learning method can be optimized is useful. We suggest that such research should be teacher-led (Churches and Dommett, 2016), because of teachers' practical insights on how learning methods could be fine-tuned to the learner group at hand. This would also help bridge the gap between L2 language learning research and instructional practices. In turn, this would enable researchers to build even more comprehensive models of L2 vocabulary learning.

Finally, our work offers novel insight into how L2 vocabulary instruction can be optimized by boosting lexical specification. Multiple-choice is already used as a digital learning tool (see Nakata, 2011). This makes the step of adding a contrasting feature easy. The next step would be to make such a tool adaptive to the learner. For instance, features could be included such as the ascertainment of an optimal difficulty level depending on the learners' language skills, or the addition of certain lexical cues for learners who require them. Such a learning tool could also be adapted differently for early and late learners, given that contrasting may show differential effects depending on the linguistic development stage of the learners (cf. Baxter et al., under review).

## Conclusion

We provide initial evidence that contrasting, a process essential for the differentiation of L2 lexical representations can, under specific circumstances, effectively be exploited in a teaching method to facilitate L2 word learning in children by sharpening their representations. Our study extends existing theories that propose a gradual lexical specification process, such as the fuzzy lexicon hypothesis (Cook et al., 2016), as well as those positing a causal link between specificity of representations and retrieval efficiency, such as the lexical quality hypothesis (Perfetti and Hart, 2002). In particular, our study takes a step further by offering insights into how the specification of L2 representations can be made more efficient for different dimensions and participant populations. As we see it, our findings offer a starting point to contribute to the successful development of more comprehensive theoretical models of L2 vocabulary learning, and to direct applications for instructional practice.

## Data Availability Statement

The raw data supporting the conclusions of this article will be made available by the authors, without undue reservation.

## Ethics Statement

The studies involving human participants were reviewed and approved by Ethics Committee Social Sciences (ECSS) of the Radboud University Nijmegen. Written informed consent to participate in this study was provided by the participants' legal guardian/next of kin.

## Author Contributions

HB, MD, MH, TD, and FL acquired the funding for the project. FL and PB designed the study. PB designed the stimuli, programmed the experiment, acquired and analyzed the data, and wrote the draft of the manuscript. PB, MD, MH, and TD developed the theoretical framework for the manuscript. MD, MH, and TD revised the early stages of the manuscript. All authors contributed to reviewing the final stages of the manuscript, approved the final manuscript, and conceptualized the study.

## Conflict of Interest

The authors declare that the research was conducted in the absence of any commercial or financial relationships that could be construed as a potential conflict of interest.
