# Supplementary material for: Contrasting Similar Words Facilitates Second Language Vocabulary Learning in Children by Sharpening Lexical Representations
Source: Front Psychol. 2021 Jul 6;12:688160. doi: 10.3389/fpsyg.2021.688160 (PMC8290082; doi:10.3389/fpsyg.2021.688160)
Supplement: Supplementary file 1 [file Data_Sheet_1.pdf]

Appendix 1

Table A.1

All stimuli used in the experiment, in the contrasted condition. The closer the value is to 1, the more similar the words are. The orthographic and semantic similarity values are not directly comparable because they are based on different metrics. The orthographically similar words are more orthographically similar than all other words, and the semantically similar words are more semantically similar than all other words.

Groups	Similarity condition	Mean orthographic similarity	Mean semantic similarity
PIGEON – POISON - PRISON	Orthographic	0.61	0.16
BEAD - BEAK - BEAM	Orthographic	0.75	0.17
PRAWN – YAWN - LAWN	Orthographic	0.65	0.11
	**Average**	**0.67**	**0.15**
TWIG – LEAF - SHRUB	Semantic	0.00	0.35
TIRE – BRAKE - GEAR	Semantic	0.13	0.34
DENTIST – PLUMBER - BUTCHER	Semantic	0.10	0.27
	**Average**	**0.08**	**0.32**
KITE – HOOVER - TRAY	Dissimilar	0.06	0.11
ARROW – DIAPER - FRIDGE	Dissimilar	0.11	0.10
CRADLE – FOAM - VEIN	Dissimilar	0.13	0.09
	**Average**	**0.10**	**0.10**

Table A.2

All stimuli used in the experiment, in the not contrasted condition. The closer the value is to 1, the more similar the words are. The orthographic and semantic similarity values are not directly comparable because they are based on different metrics. In all conditions, the words groups are dissimilar on both similarity dimensions.

**Groups**	**Similarity condition**	**Mean orthographic similarity**	**Mean semantic similarity**
PIGEON – LAWN - BEAM	Orthographic	0.11	0.08
POISON - YAWN - BEAD	Orthographic	0.06	0.07
PRISON – PRAWN - BEAK	Orthographic	0.23	0.10
	**Average**	**0.13**	**0.08**
BUTCHER – SHRUB - GEAR	Semantic	0.05	0.05
PLUMBER – LEAF - BRAKE	Semantic	0.16	0.10
DENTIST – TWIG - TIRE	Semantic	0.23	0.11
	**Average**	**0.15**	**0.09**
KITE – HOOVER - TRAY	Dissimilar	0.06	0.11
ARROW – DIAPER - FRIDGE	Dissimilar	0.11	0.10
CRADLE – FOAM - VEIN	Dissimilar	0.13	0.09
	**Average**	**0.10**	**0.10**

Appendix 2

Contact with English questionnaire

1) Do you speak English outside of school? For example, with your parents, siblings, or friends?
2) Do you watch movies or television programs in English outside of school?
3) Do you listen to music with English lyrics outside of school?
4) Do you read books or magazines in English outside of school?
5) Do you play video games in English outside of school?
6) Do you watch YouTube videos in English outside of school?
7) If you use social media, do you write, read, or listen to English? Think for example of Facebook, Twitter, and Instagram.
